# Unifying the spectrum: a framework for the meta-analysis of human skin tone scales

**DOI:** 10.1117/1.JBO.31.9.095005

**Published:** 2026-09-29

**Authors:** Amir S. Bernat, Lea Bromberg, David Sinefeld, Boaz Arad

**Affiliations:** a Voyage81 LTD, Tel-Aviv, Israel; b Jerusalem College of Technology, Applied Physics and Electro-Optical Engineering Department, Jerusalem, Israel

**Keywords:** skin tone, colorimetry, Fitzpatrick scale, monk scale, skin tone scales, skin color, colorism

## Abstract

**Significance:**

Quantification of human skin tone is fundamental to dermatology, medical imaging, computer vision, and social studies. However, the field currently lacks a standardized framework, relying on fragmented methodologies, from the Fitzpatrick skin phototype (FST) to the Monk skin tone (MST) scale. Even nowadays, this creates barriers to scientific progress and introduces systemic errors and bias in medical devices and AI fairness.

**Aim:**

The aim of this study is to establish a meta-analytic framework, termed a “scale of scales,” to critically assess and quantitatively compare the interoperability and accuracy of prevailing skin tone quantification systems.

**Approach:**

We utilized the International Skin Spectra Archive (ISSA), composed of 15,256 spectral reflectance measurements from a globally diverse population, as a biological baseline. We overlaid the categories of major verbal, physical, and digital scales onto this dataset to evaluate their coverage and fidelity.

**Results:**

Our analysis reveals that widely used scales such as FST and MST fail to capture the continuous, multidimensional nature of human pigmentation. Furthermore, physical targets such as the Pantone skintone guide exhibit significant metameric failure, rendering them unreliable under varying illumination.

**Conclusions:**

Current subjective and categorical proxies are insufficient for robust scientific application. The field must transition toward a unified, data-driven framework rooted in spectral reflectance data to ensure interoperability and inclusivity in medical and technical applications.

## 1 Introduction

Human skin pigmentation is a continuous evolutionary heritage adapted to environmental conditions, not a set of discrete categories.^1^ Quantification of human skin tone is a critical requirement for a variety of disciplines, ranging from medical diagnostics and pulse oximetry^2^^–^^5^ to AI fairness,^6^^,^^7^ cosmetic science,^8^ and anthropology.^1^ During the COVID-19 pandemic, pulse oximeter devices were found to overestimate the saturation level of dark-skinned patients,^2^^,^^9^^,^^10^ resulting in occult hypoxemia. Established and emerging imaging modalities such as optical coherence tomography (OCT),^11^ photoacoustic imaging,^12^^,^^13^ and spatial frequency domain imaging (SFDI),^14^ have also shown sensitivity to skin tone, mainly due to differences in the concentration of melanin in the epidermis. The relation and effect of tissue content and melanin concentration were shown in lab tests, and computational modeling using Monte Carlo simulations,^15^ and also in phantom models.^4^ Despite this ubiquity, the field lacks a standardized framework.^3^^,^^16^ This fragmentation impedes robust comparison of research findings, prevents the aggregation of data between studies, and complicates the regulation of clinical devices and commercial products.

The classification of human skin tone has a vast historical lineage.^17^ Modern applications have necessitated standardization, leading to the development of the FST scale,^18^ the Macbeth ColorChecker,^19^ and the individual typology angle (ITA)^20^ to name a few. More recently, the landscape has expanded to include the MST scale^21^ and the Pantone skintone guide (PSTG).^22^^,^^23^ Currently, there is no method to facilitate cross-disciplinary research between disparate scales and fields, such as skin graft matching^24^ and social bias evaluation,^25^ representing two extremes of the spectrum.

Current methodologies generally fall into three categories: verbal classification scales (e.g., FST), which tend to use an ordinal level of measurement and few categories. Reference targets (physical or digital) are used for visual matching and are often designed to be an interval level of measurement and contain a larger number of categories. Imaging or spectral scales (e.g., ITA and MI), which provide ratio-level data, thus providing a continuous scale. Previous work has highlighted recurring issues across these categories, including disparity in the representation of dark skin tones, poor prediction of skin colors, and nonmonotonic and inconsistent intervals.^26^^–^^28^

This paper establishes a comprehensive meta-analytic framework to critically assess and compare these systems. Using the ISSA dataset^29^ as a biological baseline for human skin color, we provide a data-driven evaluation of the color accuracy and interoperability of current skin tone scales. We expand both the number of scales evaluated and the evaluation criteria compared to similar critical evaluations.^3^^,^^27^^,^^28^^,^^30^ This approach differs from many recent publications that compare different scales with skin impressions in uncontrolled settings and evaluate based on the level of agreement between the assessors and the measurement devices.^9^^,^^26^^,^^31^ Our goal is to focus our evaluation on tools that can be used in a general setting; we therefore, did not include high-performance liquid chromatography (HPLC) or tissue staining technology^32^^–^^34^.

## 2 Methodology and Data

To evaluate and compare the diverse landscape of skin tone scales, we utilized the ISSA as a representative biological baseline. This multicultural human skin phenotype dataset consists of 15,256 reflectance spectra with 10 nm resolution, allowing us to simulate how different scales would interact with human skin physics. Data were collected from 2113 subjects in eight countries, ensuring a wide representation of gender, age, and ethnicity as shown in Fig. 1.

**Fig. 1:**
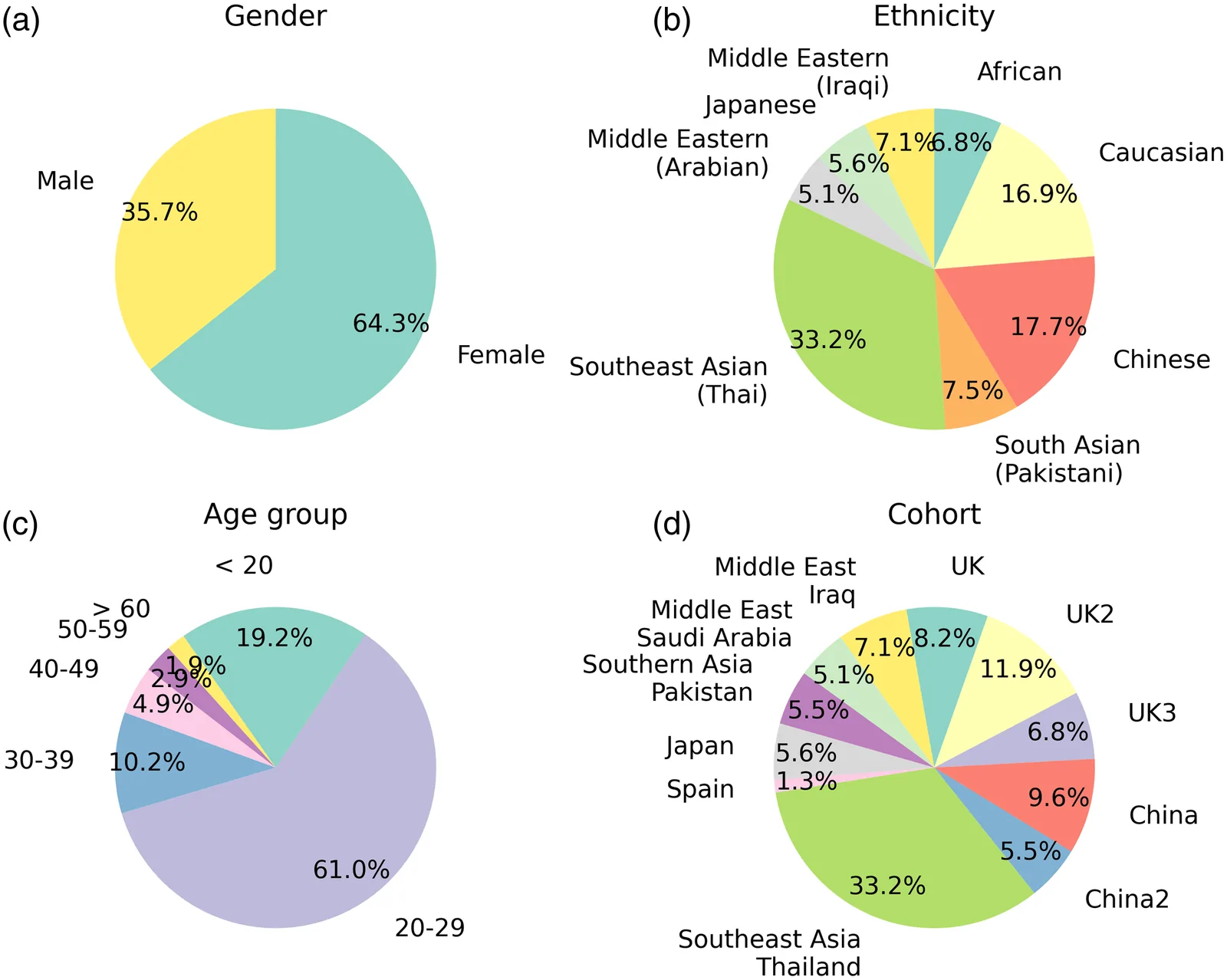
Demographic distributions of the ISSA dataset, illustrating: (a) gender, (b) ethnicity, (c) age group, and (d) cohort of origin (comprising sub-cohorts from China, Iraq, Japan, Pakistan, Saudi Arabia, Spain, Thailand, and the United Kingdom).

Although the dataset includes measurements from several body locations, this study focuses strictly on facultative skin tones derived from forehead measurements.

Although the baseline data within the ISSA framework has demonstrated long-term stability and cross-site reliability over its collection history,^35^ it does not explicitly account for short-term ambient or behavioral fluctuations. Physiological factors, such as a subject’s hydration status, localized perspiration, and blood pressure, alongside environmental variables such as ambient and room temperatures and humidity, are well-documented sources of transient artifacts in superficial microvascular skin perfusion.^36^ However, when analyzing aggregated cohort data (i.e., at the population level), these individual physiological variances are unlikely to significantly change the aggregate data, making the collective dataset resilient to these localized fluctuations and yielding a highly reliable baseline phenotype compared to isolated, single-subject measurements.

We present the ISSA subjects and the evaluated scales using the CIE-LCH color space (Lightness, Chroma, Hue). This transformation highlights the differentiation between the colors and undertones (red-yellow hue directions, referred to as an undertone in the cosmetics industry^37^) of different ethnic groups,^38^ as visualized in Fig. 2. Previous work has evaluated undertone on uncalibrated images “in the wild”^39^ and not based on spectrally derived colors. The Caucasian population can be seen to differ along the Hue axis from other ethnicities with a similar level of lightness.

**Fig. 2:**
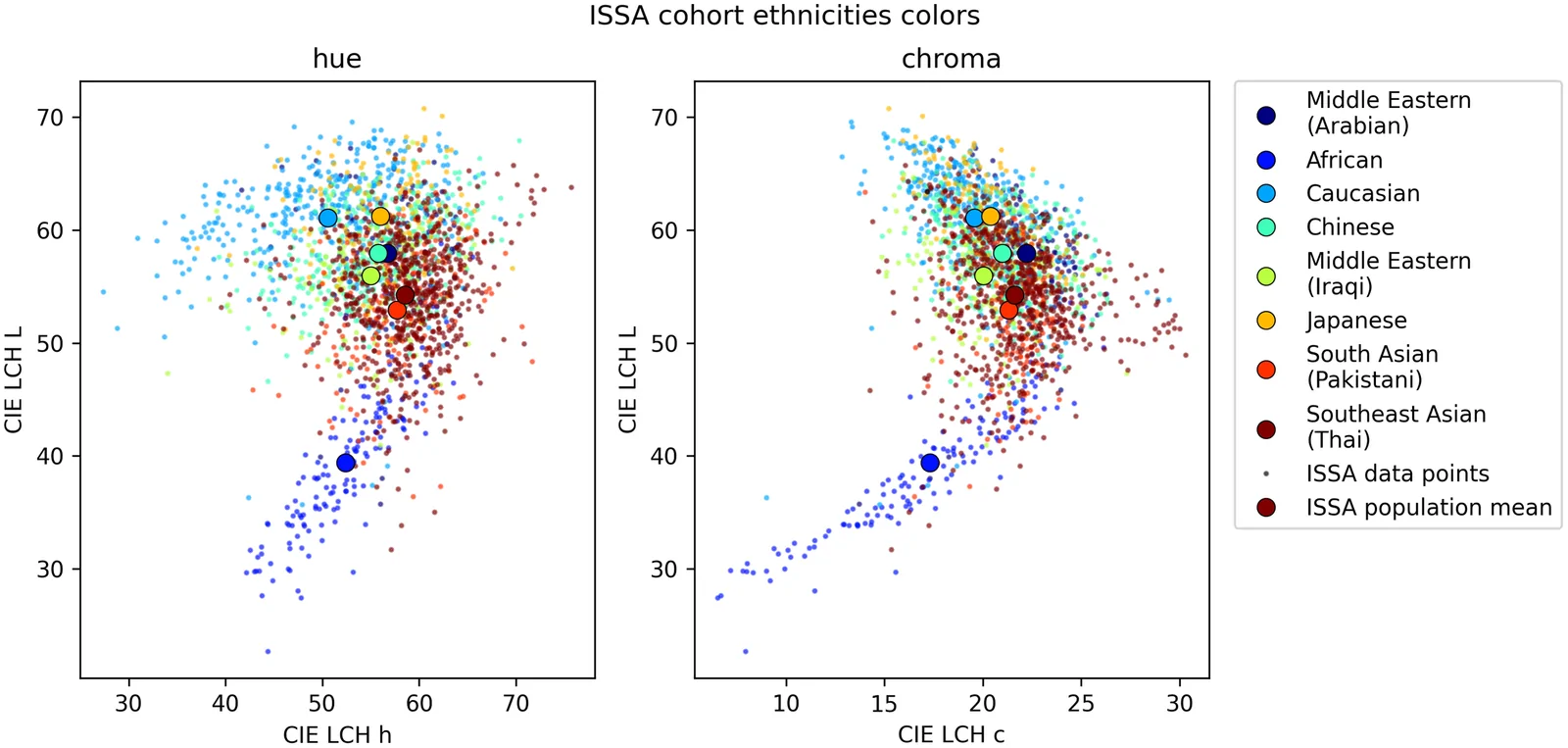
CIE-LCH color values of forehead measurements in the ISSA dataset, divided by indicated ethnicity in the dataset. Solid points mark each ethnicity’s mean color values.

To perform the meta-analysis, we overlaid the color categories of each reviewed scale onto the ISSA population data. The color values for these scales were acquired using tools appropriate for their format:

• **Physical Targets:** Measured using an X-Rite CI62 sphere-based spectrometer set to D65 illuminant, and specular component excluded
• **Digital Scales:** Provided numerical values when possible, otherwise programmatically extracted from official PDF documents or images
• **Verbal Scales:** We only discuss the FST scale in this context. The FST lacks standardized color values; a representative image was selected as the source for analysis

Finally, for each scale, we calculated an annual citation rate based on Google Scholar data to quantify its acceptance and prevalence in the literature. This metric reflects prevalence and engagement rather than endorsement. We did not distinguish critical citations from supporting ones, nor did we filter to reflect citing publications that addressed skin tones specifically.

## 3 Results

### 3.1 Verbal and Classification Scales

FST is clearly the most cited scale in our review (Annual Citation Rate 150). Despite its dominance, the FST lacks standardized color values; widely used depictions are inconsistent representations.^16^^,^^40^ When overlaid on the ISSA data (Fig. 3), a stark disparity is evident: Phenotypes V and VI exhibit a difference of ∼30 CIE-L units, whereas Phenotypes I to IV are spaced apart at ∼10 units. Furthermore, phenotypes I and II are noticeably lighter than the lightest facultative skin tones in the ISSA dataset.

**Fig. 3:**
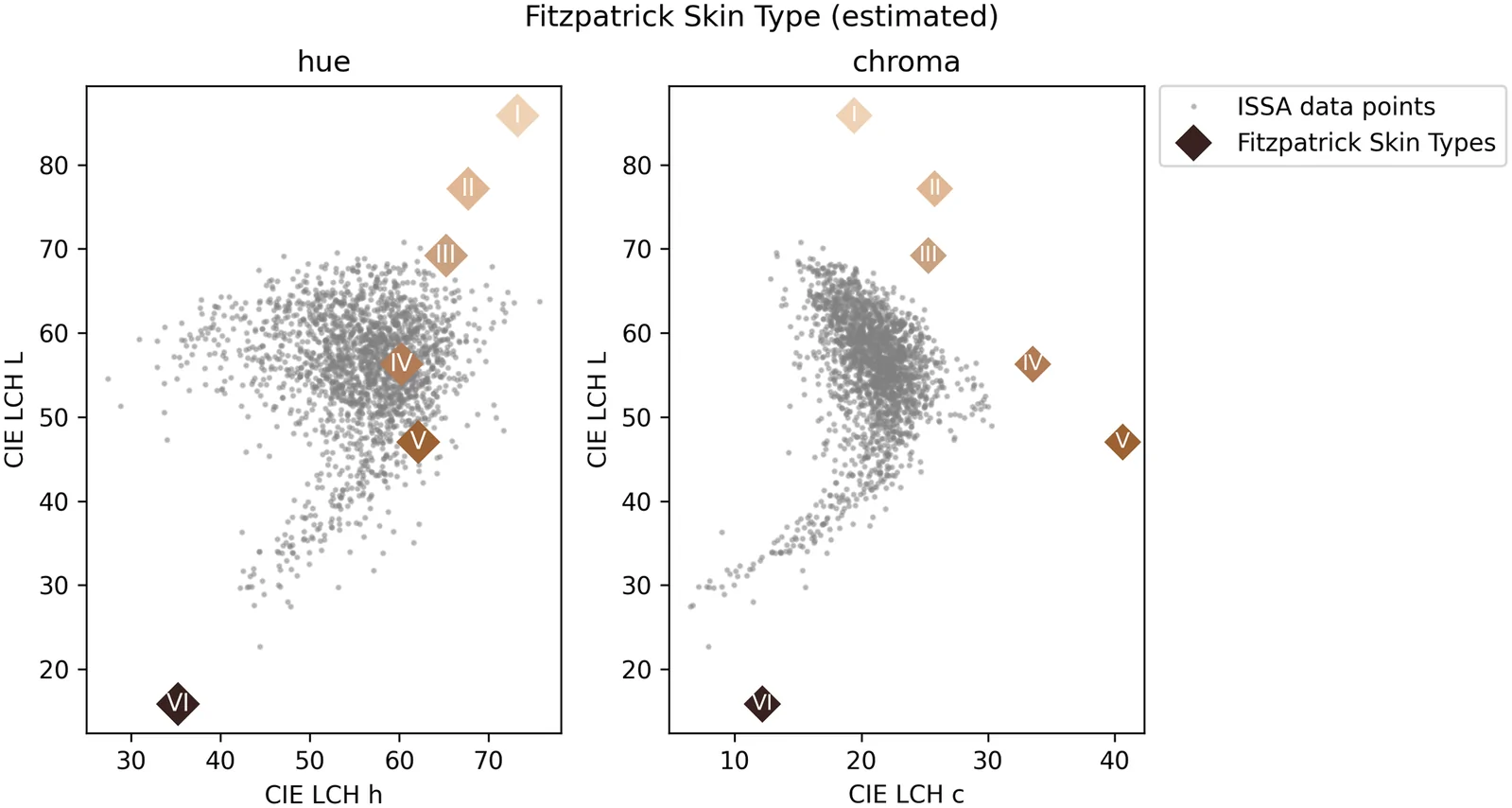
Fitzpatrick skin phototype. It can be seen that phenotypes I and II are lighter than the ISSA data; phenotype VI is darker and unevenly spaced compared with previous phenotypes. Color values are based on common color reproductions; no agreed common values exist.

### 3.2 Physical Reference Targets

The first physical skin tone targets in modern times were the von Luschan skin color tiles^41^ using 36 opaque glass tiles, which have been out of production for over 70 years and are therefore not included here. Currently, among physical targets, the PSTG is the most widely accepted, with the post-2023 version Fig. 4 offering 138 chips. Although the updated guide improves the coverage of red undertones compared to its predecessor, it introduces a noticeable gap in coverage around CIE-h=55° and CIE-L between 35 and 70 units.

**Fig. 4:**
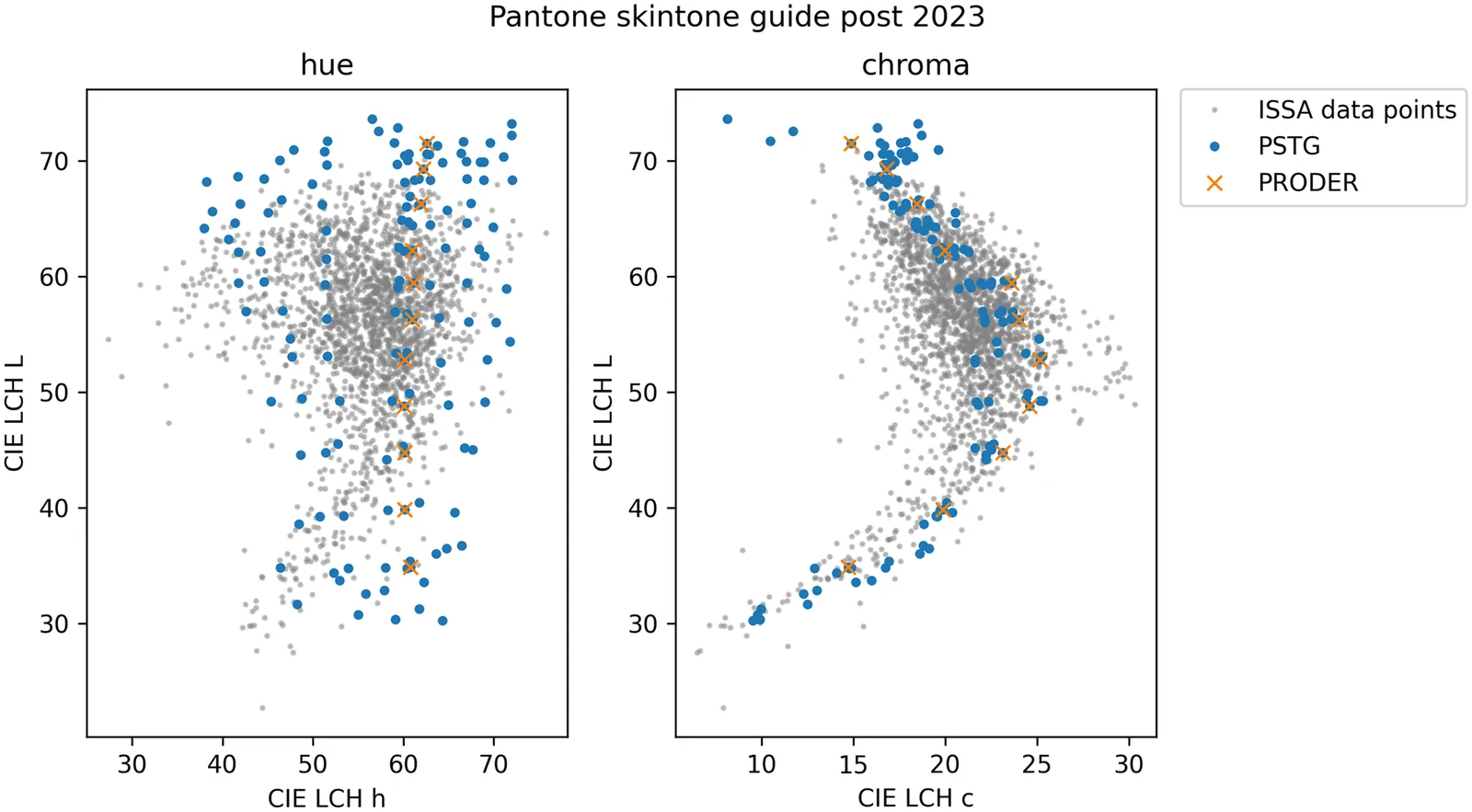
Pantone skin tone guide (post-2023) with 138 points. Note the division in the hue point cloud around CIE-h=55° and CIE-L between 35 and 70 units. orange “×” are used to mark selected PSTG patches used in the PRODER scale.

Crucially, our spectral analysis reveals that many of the PSTG chips suffer from metameric failure, as do the IT8.7 skin patches. Although it is advised to use the PSTG under D65 illumination, if end users do not strictly adhere to these guidelines, significant errors might occur. In a scenario in which two clinicians evaluate skin grafts under warm and cool lighting, the matches can diverge greatly. Simulations under Illuminant A show that many of the chips shift color drastically compared to how actual human skin would appear under the same lighting. As shown in the spectral curves (Fig. 5), this is because many PSTG chips do not exhibit the characteristic tapering of human skin reflectance beyond 620 nm. A specific use case of the PSTG is the PRODER scale,^42^ where a subset of 11 color chips from the full guide was selected, having even ITA spacing and all with 1 Y undertone.

**Fig. 5:**
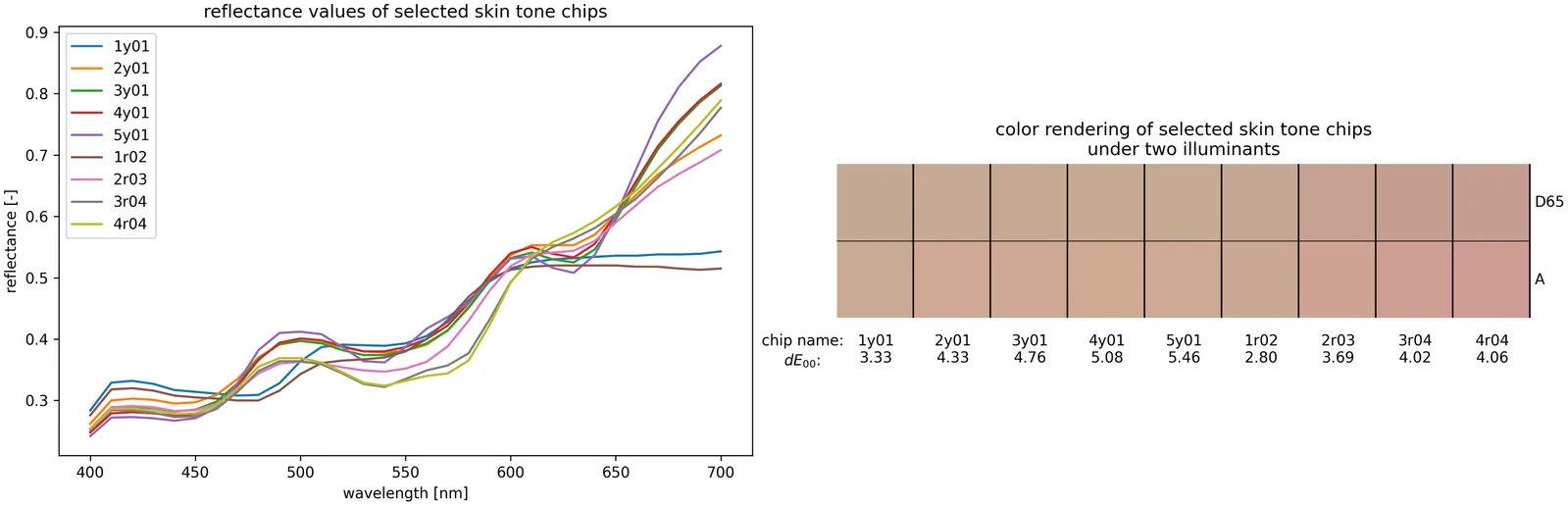
Example for color rendering of Pantone SkinTone Guide^22^ patches are rendered from reflectance under illuminants D65 and A. Here, color chips 1Y01 and 1R02 taper off past 620 nm, as would human skin, while other color chips seen here show a considerable rise in reflectance in that region. This difference causes the stark visual difference in the appearance of chips under illuminant D65 and A, which users should be mindful of.

Additional physical charts reviewed, such as the Spyder Checkr^43^ and X-Rite ColorChecker SG,^44^ lack sufficient categories below CIE-L 50 and are frequently lighter than the ISSA data. Several common issues can be seen in Fig. 6, primarily:

• Other than the IT8.7 chart from Wolf Faust^45^ most charts are very lacking in examples of skin tone charts darker than CIE-L 50
• Most charts present numerous categories considerably lighter than the ISSA data
• Most targets do not specify use instructions, what reference illuminant should be used, whether they were designed for use with facultative or constitutive skin areas, etc.

**Fig. 6:**
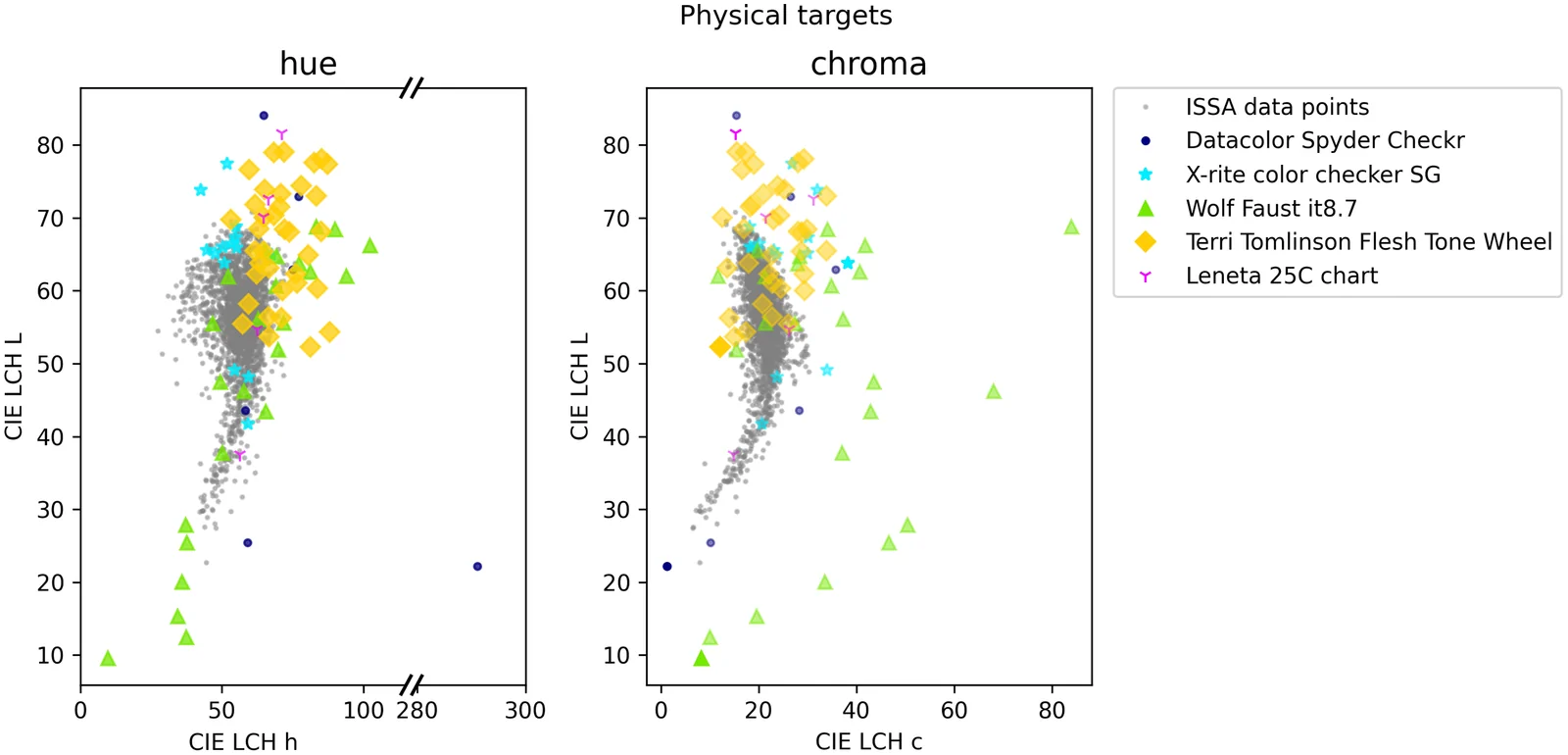
Several physical color targets overlayed on the ISSA data. Shown here are the Spyder Checkr from Datacolor,^43^ Color checker SG from X-Rite,^44^ IT8.7 from Wolf Faust,^45^ Flesh Tone Color Wheel from Terri Tomlinson,^46^ 25C chart from Leneta.^47^

### 3.3 Digital Color Scales

The transition to digital workflows has prompted the development of print- and screen-based scales, yet they suffer similar limitations to their physical counterparts. The MST scale^21^ Fig. 7, originally developed for social evaluation, has gained significant traction in computer vision and AI fairness, endorsed by Google.^48^ Despite its prevalence (Annual Citation Rate 50), it was criticized for several flaws: The 10 categories of the scale are unevenly spaced, nonmonotonic, and poorly predictive of actual skin spectra.^27^^,^^28^^,^^49^ Furthermore, the lightest and darkest MST color values are out of gamut for standard commercial printing, this forces printers to perform gamut mapping (compressing colors), which fundamentally alters the scale’s appearance depending on the specific printer driver used, affecting consistency between research sites.

**Fig. 7:**
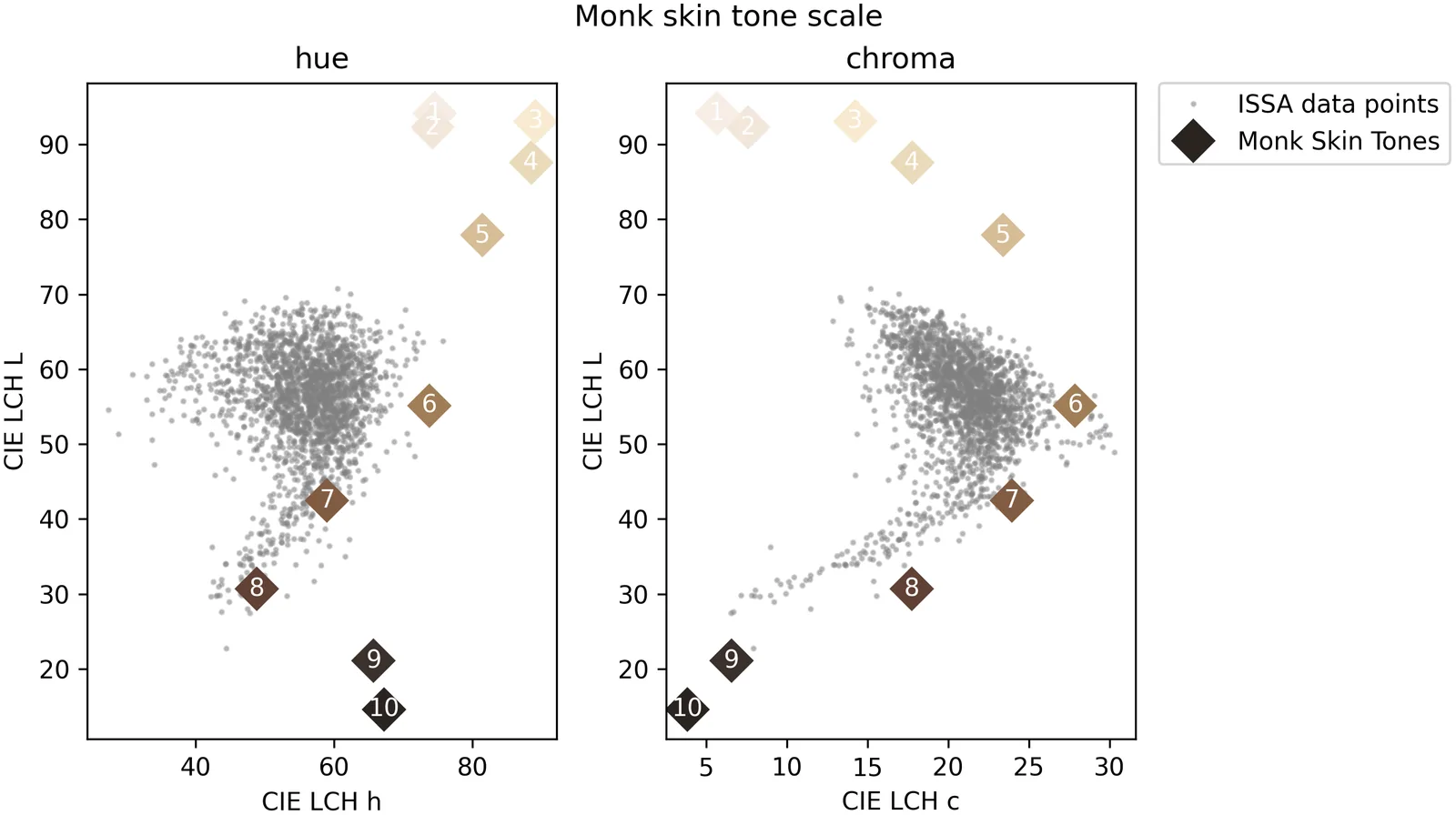
Monk scale overlayed on the ISSA data. It can be seen that only three categories match well with the ISSA data, categories that are very light CIE-L>90, and very dark CIE-L<20 can be out of gamut for many printers.

The digital scale with the most categories is the L’Oréal New Skin Color Chart, Fig. 8. This scale was originally described and published as having 72 colors,^8^ and with values that should match the ISSA data; however, available sources only provide 66 categories with 11 lightness and six undertone values,^50^ less than half of which match the ISSA data.

**Fig. 8:**
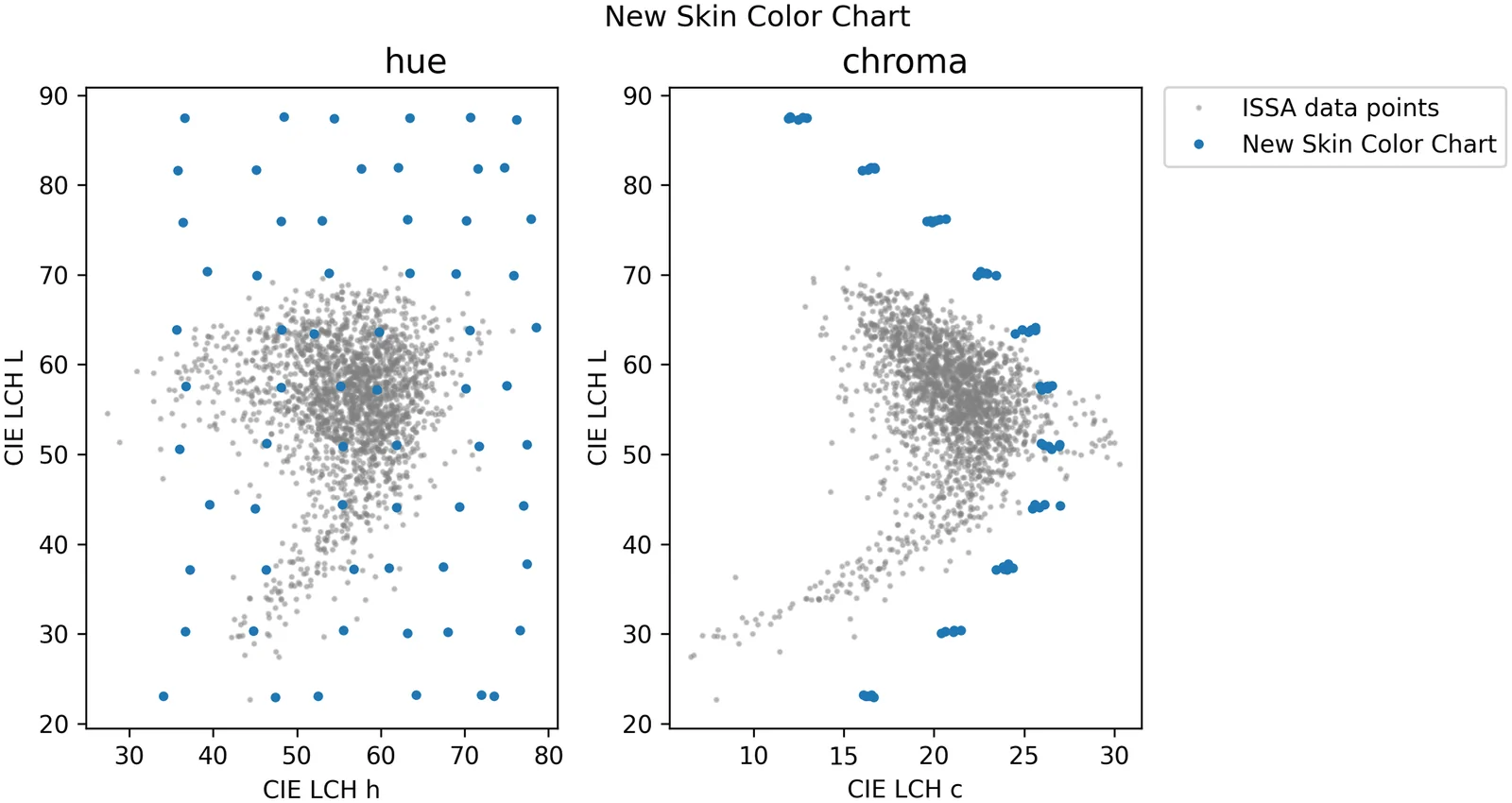
L’Oréal New Skin Color Chart^8^ with 66 colors derived from image data.^50^ The original publication had 12 lightness values, which matched ISSA values much better; however, only a partial section was published. Most of the colors in the image data do not match up with the ISSA data.

Other tested scales are shown in Fig. 9, where most also suffer from similar issues, with only the scales derived from spectra^30^^,^^38^ being a positive outlier. The Colorimetric Skin tone Scale (CST) and the skin color clusters (SCC) both scored a low annual citation rate because they were published less than a year before this review. The CST scale design procedure considers both skin colors of the face and the back of the hand, which may explain the design selection to favor the red over yellow undertone. The SCC scale^38^ assigns only six clusters in five different lightness levels, which match the ISSA data well.

**Fig. 9:**
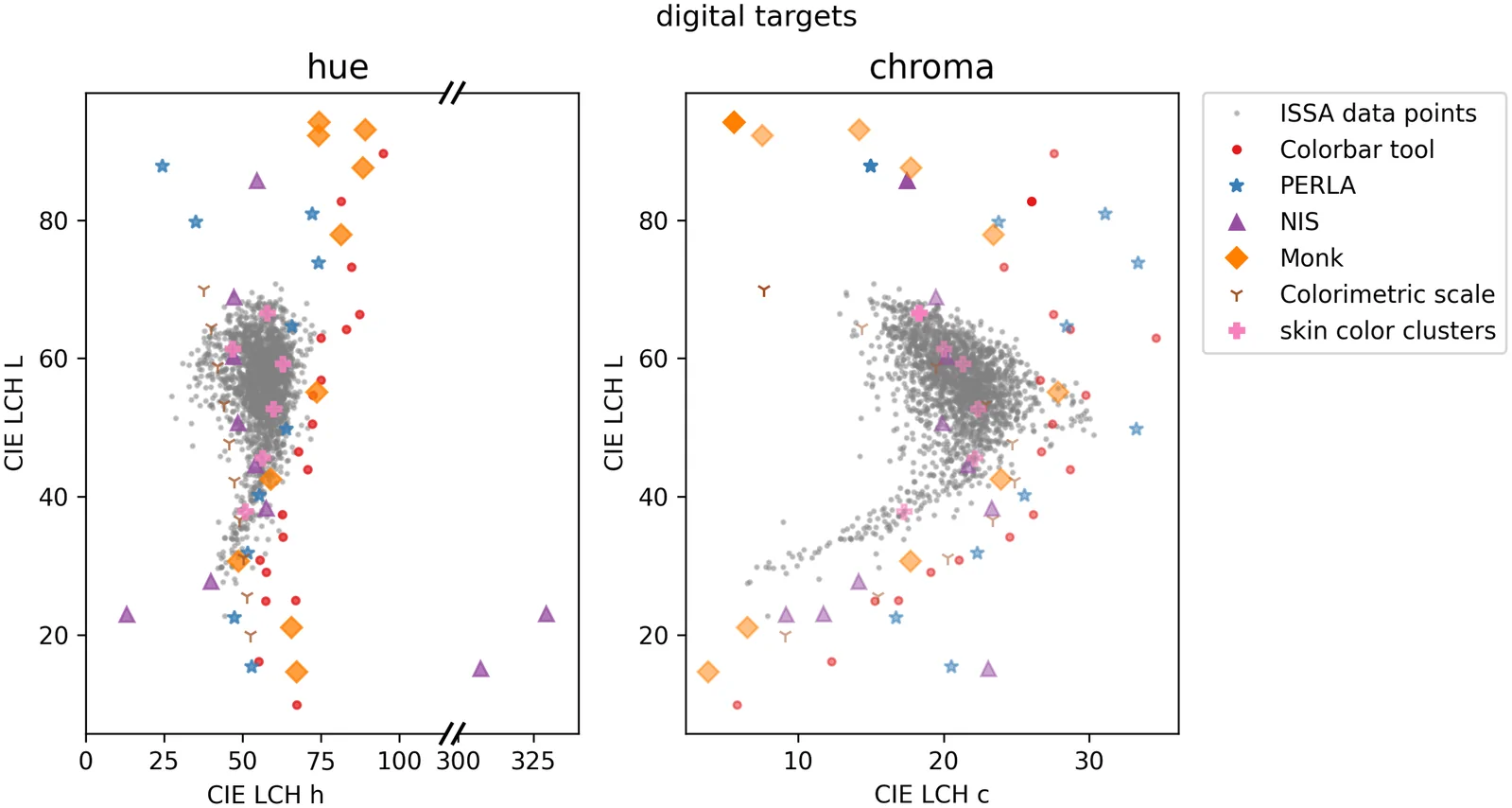
Additional digital scales are overlaid on the ISSA data. Shown here are the colorbar tool,^51^ PERLA scale,^52^^,^^53^ NIS (Massey-Martin),^54^ Monk,^21^ colorimetric skin tone, and ^30^ skin color clusters^38^ scales.

Common issues seen in the presented digital scales include:

• Poorly predictive skin tones
• Nonmonotonic and uneven spacing between categories
• Unrealistic color cast
• Lack of use and reproduction requirements

### 3.4 Imaging and Spectral Scales

Metric-based scales derived from measurement devices provide continuous ratio level of measurement data, but face standardization hurdles. ITA was developed to track suntanning pathways in the Caucasian population and is calculated using CIE-L and CIE-b.^20^ However, this definition makes ITA agnostic to hue variations along the CIE-a axis. Consequently, ITA planes were criticized for not depicting changes in Asian populations, specifically the Chinese population.^55^ As visualized in Fig. 10, the difference between Caucasian and Asian populations is primarily along the red-yellow axis. Defining the scale without considering this difference results in coarse separation and significant overlap between ethnicities with a higher average H angle (yellower) skin.

**Fig. 10:**
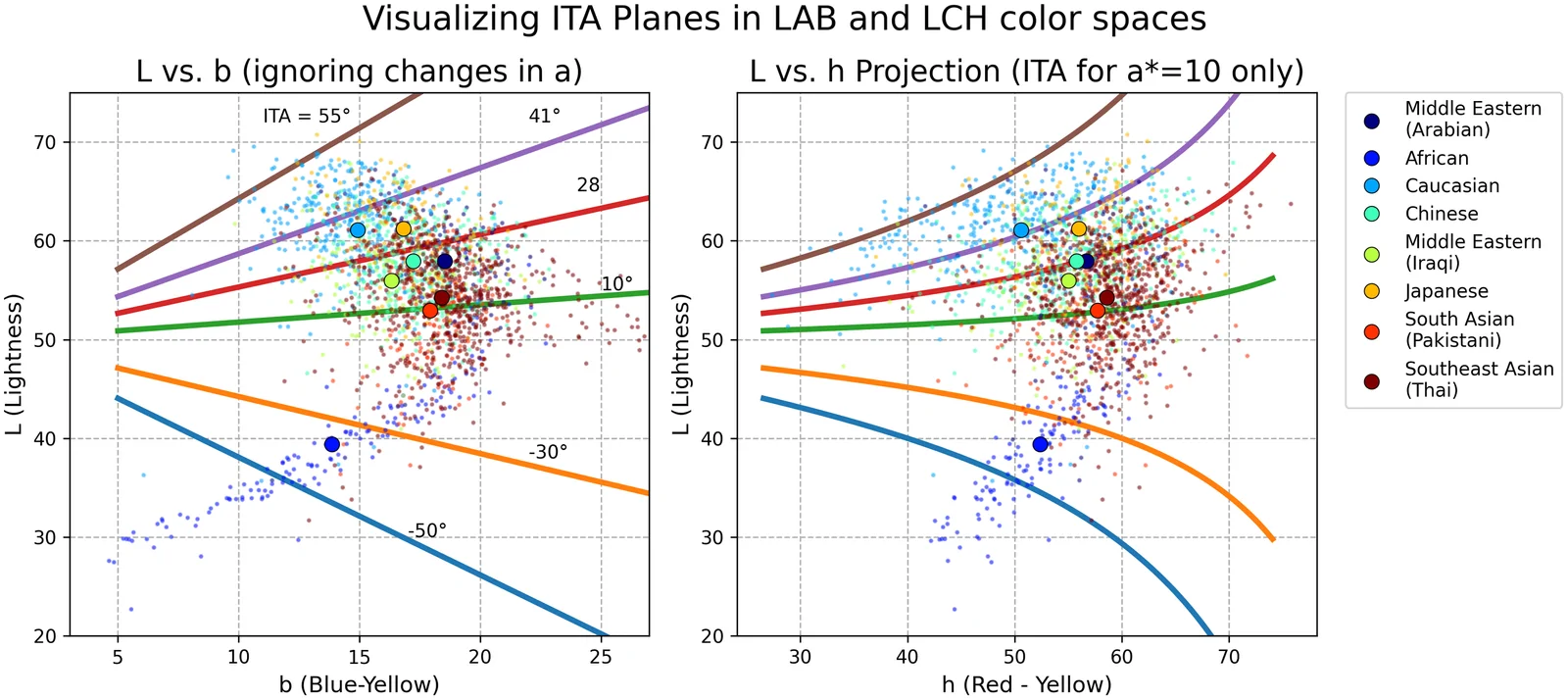
Depiction of ITA curves over the ISSA dataset L-b (left) and L-h (right) panels with ethnicities shown in different colors. Straight lines in the L-b panel transform to curves in the L-h. The rendering of curves in the L-h panel is done for CIE-a=10.

As shown in Eq. (1), the metric is derived using two specific colorimetric values, where:

• L Lightness value
• b Blue-Yellow value
  $$\mathrm{ITA}{}^{\circ}=(\arctan(\frac{L-50}{b}))\times \frac{180}{\pi }.$$ (1)

Melanin (MI) Fig. 11 and Erythema (EI) Fig. 12 indices^56^ provide biological fidelity, referencing approximate tissue content rather than surface color. However, these indices lack a single agreed-upon definition,^3^ values are defined by proprietary algorithms and specific wavelengths (e.g., narrow-band versus broad-band filters, specific band selection) that vary between device manufacturers. Although they are considered to be a “gold standard” for accuracy, the lack of a unified spectral definition limits their interoperability across different instruments and scales. Here, we use the definition provided for the DSM III (Cortex Technology) device for both MI Eq. (2), and EI Eq. (3).

$$\mathrm{MI}=\log_{10}(\frac{1}{R_{655\;\mathrm{nm}}})\times 100,$$ (2)

$$\mathrm{EI}=\log_{10}(\frac{R_{655\;\mathrm{nm}}}{R_{568\;\mathrm{nm}}})\times 100,$$ (3)

where the reflectances are linear interpolations of reflectance values from the nearest reported wavelengths.

**Fig. 11:**
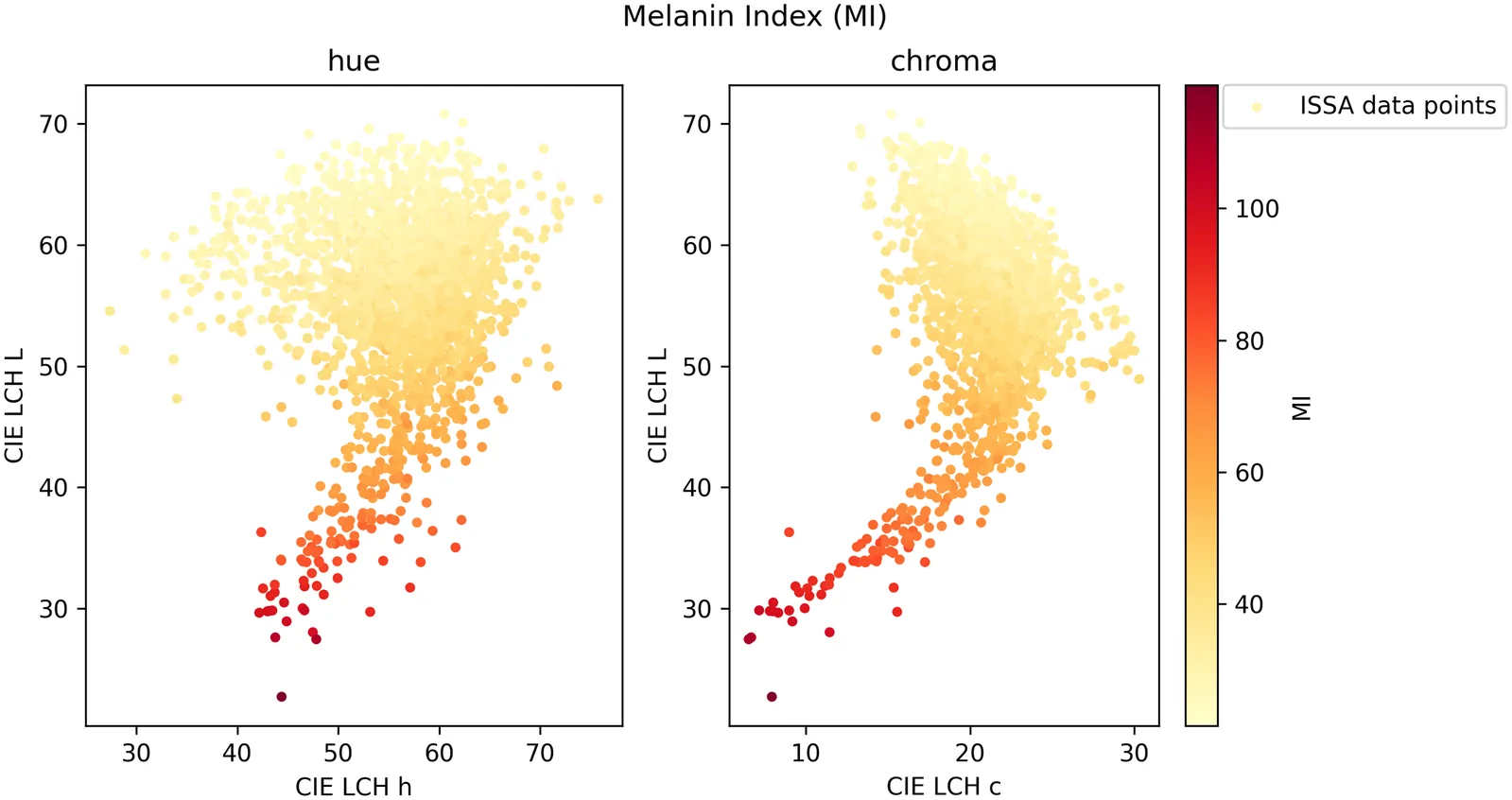
Melanin index values are presented as color codes over CIE-LCH space. It can be seen that lightness value strongly correlates with MI.

**Fig. 12:**
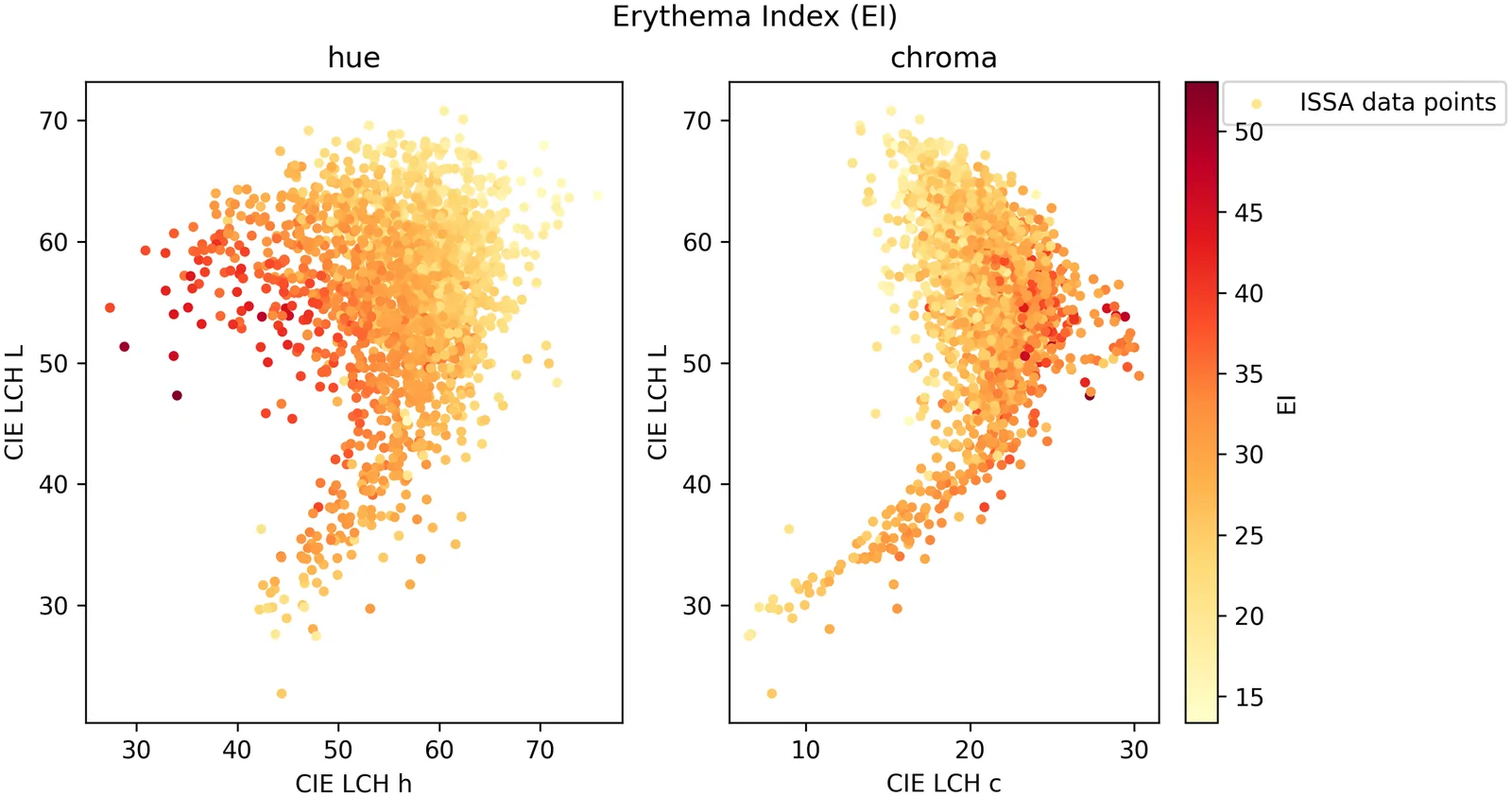
Erythema index values are presented as color codes over CIE-LCH space. It can be seen that the EI values sharply rise along the left side of the CIE-h. Lower CIE-h values indicate subjects with redder skin tones.

### 3.5 Comparison Tables

To provide a comprehensive overview, we aggregated the reviewed scales into a single meta-analytic framework (Tables 1–3). The scales are sorted by their annual citation rate and are split into tables based on the existence of source publications to be referenced. For each scale we present the intended matching tool to be used for evaluation, the number of categories and level of measurement, the scale’s original intended goal, annual citation rate in Google Scholar per January 10th 2026, any comments about the scale. Table 1 depicts scales that are well discussed in academic literature, Table 2 depicts scales that are discussed in academic literature, but we have not identified a specific publication to reference them, Table 3 depicts scales that have some skin tone information but are mainly used in other fields.

**Table 1:** Collection of scales reviewed in this study, which are designed as skin tone evaluation tools and have specific citation sources. Level of measurement abb. N – nominal, O – ordinal, I – interval, and R - ratio.

Scale	Matching Tool	Categories (level)	Intended Goal	Annual Citation Rate	Comments
FST^18^	Questionnaire	I-VI (O)	UV sensitivity	150 since 1988	Disparity to dark skin tones, No numeric color values
PERLA^52^	Visual	8 (O)	Social studies in South America	100 since 2014	Uneven spacing, unrealistic skin tones
MST^21^	Imaging	10 (O)	Skin tone evaluation in social studies and computer vision	50 since 2023	poorly predictive of skin tone
PRODER^42^	Visual	11 (I)	Skin tone evaluation in social studies in Mexico	41 since 2025	A very recent addition, only matches yellow undertones
ITA^20^	Spectral + Imaging	Float / 6 (I)	Suntanning pathways	20 since 1991	When grouped to 5 to 6 categories dark skin disparity
Eumelanin Human Skin Colour Scale^57^	Spectrometer + Melanometer	5 (R)	Tissue content	15 since 2022	This scale is a division of MI to 5 categories
NIS (Massey-Martin)^54^	Memory	10 (O)	Immigrant ethnicity categories	11 since 2003	Darkest categories show a strong blue cast
New Skin Color Chart^8^	Visual	66 (I)	Cosmetics	4 since 2007	Over half of the categories do not match skin tone
Colorimetric skin tone scale^30^	Visual + Imaging	10 (I)	Homeland Security	3 since 2025	A very recent addition, color values are averaged from face and hand data
Skin color clusters^38^	Spectral + Imaging	6 (I)	Cosmetics development	None in the 4 months prior to this review	A very recent addition, There are 5 lightness levels, one split to two undertones

**Table 2:** Collection of scales reviewed in this study for which no specific publication was found; instead, a Google Scholar keyword search was used to estimate the annual citation rate.

Scale	Matching Tool	Categories	Intended Goal	Annual Citation Rate	Comments
Melanin and Erythema index^56^	Spectrometer + Melanometer	Float (R)	Tissue content	100 since 1980s	Requires dedicated expensive devices
Pantone Skin Tone Guide (pre-2023 / post-2023)^22^	Visual	110/138 (I)	Physical rendering of human Skin	31 since 2012	Sensitive to lighting changes
Leneta chart 25C^47^	Visual	5 (O)	Evaluation of cosmetics products	NA	Uneven spacing, non-monotonic, dark skin disparity
Terri Tomlinson flesh tone color wheel^46^	Visual	36 (O)	Makeup matching	NA	Disparity to dark skin tones

**Table 3:** Collection of scales reviewed in this study, these color charts are not dedicated for use specifically for skin tone evaluation; for that reason, we omitted the annual citation rate in this table.

Scale	Matching Tool	Categories	Intended Goal	Comments
ColorChecker SG skin tones^44^	Digital cameras	12 (N)	Digital camera calibration	Mostly cultural heritage
Spyder color checker skin tones^43^	Digital cameras	8 + 2 (O)	Digital camera calibration	lightest and darkest are very much removed from ISSA data
IT8.7^45^	Film Camera / scanner	24 (N)	Camera and scanner calibration	Only 15 of the 24 patches match human skin color, colors are noticeably more vibrant

## 4 Conclusions

The comprehensive meta-analysis presented in this study reveals a fundamental disconnect between the prevailing methods of skin tone quantification and the biological reality they aim to represent. By benchmarking the most widely used verbal, physical, and digital scales against the ISSA dataset, we have demonstrated that current tools, ranging from the Fitzpatrick Skin Phototype to the Monk Skin Tone scale, do not capture the continuous, multidimensional nature of human pigmentation adequately. This fragmentation is not merely a taxonomic inconvenience it introduces significant systemic error into medical diagnostics, computer vision benchmarks, and social equity evaluations. To bridge this gap, the field would benefit from transitioning from subjective, categorical proxies toward a unified, data-driven framework rooted in spectral reflectance data.

### 4.1 Guidelines for Scale Selection and Design

The fragmentation of skin tone analysis is often driven by the mismatch between research goals and tool capabilities. To aid researchers in selecting the appropriate scale and to guide the development of future systems, we propose the following framework. Researchers should evaluate candidate scales against four primary questions:

1. **Granularity & measurement level:** Does the study require coarse grouping (e.g., social bias studies) or fine-grained discrimination (e.g., medical diagnostics)?
2. **Dimensionality:** Skin tone is multidimensional. Would the study suffice with scales that track solely lightness, or would they be insufficient for populations where variation is also driven by hue (undertone)?
3. **Evaluation method:** Does the scale align with the collection method (visual inspection versus instrumental measurement)?
4. **Field norms and limitations:** What scale is the norm in the field? What are the advantages and shortcomings of the scale?

Based on our meta-analysis, we advise that future skin tone scales adhere to the following design principles:

• **Physical plausibility:** Categories must strictly match human skin spectra, and subsequently, color. Be mindful of selected body locations, facultative and constitutive skin tone, sensitivity to illumination settings, and even noncolor cues such as texture and gloss. Scales containing out-of-gamut colors or metameric failures introduce significant error.
• **Top-down value design:** Provide different levels of measurement color values.
  ◦ Continuous spectral definitions, with ratio-level spectral data. This can be related to biologically relevant properties, such as MI or EI.
  ◦ Monotonic interval categories, spaced evenly and ordered monotonically to allow for valid statistical analysis and which could be rendered on a screen or printed.
  ◦ Ordinal verbal categories, with both verbal descriptors and numerical values of color values for rendering.
• **Visual examples and accuracy metrics:** Developers should provide specific image examples and accuracy metrics (mean/max dE) to ensure consistent reproduction and use.

### 4.2 Guidelines for Using the Comparisons Presented in This Paper

To provide the reader with a clearer understanding of how to use the meta analysis in this paper, we add three examples that show how to evaluate the scales’ match to specific, common use cases.

Large-scale, remote digital questionnaires. When deploying crowd-sourced or remote epidemiological evaluations where physical hardware is unavailable, investigators may choose to rely on low-granularity verbal scales that simplify data collection, such as the classic FST. Researchers should recognize that the FST only provides coarse granularity (six categories); moreover, as the FST is an ordinal level of measurement, it is problematic to treat these categories as linear intervals. Problems occur, for example, when plotting FST as a uniform scale: implying that phenotype IV has twice the pigment volume or dark-skin characteristics of the phenotype II. This can be seen in the very detailed work by Lipnick et al.,^9^ where the FST, among other scales, is presented as interval, which highlights the scale’s nonuniform spacing. Common depictions of the FST scale have no undertone distinctions. Despite the aforementioned challenges, this scale is of very common use and is considered the norm in many fields.
In-person multi-site clinical field trials. For controlled field studies featuring localized physical assessments, a physical tool featuring an interval-level design, such as the PRODER scale (a selected subset of the Pantone skintone guide with 11 categories), is highly advantageous. By utilizing standardized physical patches, cross-site observers achieve predictable, evenly spaced perceptual steps. Researchers should remain cautious, however, if their cohort contains a substantial European demographic, as the PRODER framework is optimized for populations with predominant yellow/warm undertones and lacks coverage for distinct red/cool undertones (see Fig. 4). The PRODER scale is relatively new, and despite a high citation rate, it is still limited in adoption by many scientific disciplines.
High-precision laboratory diagnostics. In scenarios requiring numeric physical quantities, such as pulse oximetry validation or melanin-absorption modeling, researchers should consider foregoing categorical approximations entirely in favor of ratio-level continuous metrics such as the Melanin Index (MI) and Erythema Index (EI). Utilizing spectrally derived indices allows for linear correlations with tissue chromophores.^3^^,^^34^^,^^56^ Although this methodology represents the gold standard for clinical precision, it requires specialized, calibrated instrumentation and trained clinical personnel, rendering it less practical for rapid, large-scale, field-based collections or for not in person studies. Combining MI and EI provides the multidimensional coverage for both lightness and undertones. Because these scales were defined differently by different device manufacturers, it is imperative to note the computation that is carried out by the device in use. These scales are commonly used in more medically oriented studies.

### 4.3 Summary

The quantification of human skin tone has historically relied on a fragmented landscape of subjective questionnaires and disjointed color targets. This study demonstrates that the most prevalent scales, including Fitzpatrick and Monk, often fail to accurately represent the biological reality of human skin spectra, particularly for darker phenotypes and hue-variant populations.

To “unify the spectrum,” the field should transition toward a data-driven framework rooted in biological plausibility. Using comprehensive datasets such as ISSA as a common baseline, we can establish interoperability between medical, social, and technical fields, ensuring that skin tone analysis is scientifically robust and inclusive.

## Data Availability

Code and data supporting the findings of this study are publicly available on GitHub at https://github.com/Amirbernat/Unifying-the-spectrum.
